# Genetic relationship between Sjögren's syndrome and abdominal aortic aneurysm: insights from a European population's genome-wide association analysis

**DOI:** 10.3389/fcvm.2025.1554991

**Published:** 2025-09-24

**Authors:** Yunanji Zhou, Hao Xiong, Qinghua Luo, Zhaohui Ding, Jun He, Lihua Wang

**Affiliations:** ^1^ Qi Huang Chinese Medicine Academy, Jiangxi University of Chinese Medicine, Nanchang, Jiangxi, China; ^2^ School of Clinical Medicine, Jiangxi University of Chinese Medicine, Nanchang, Jiangxi, China; ^3^ Pulmonary Disease Department, Affiliated Hospital of Jiangxi University of Traditional Chinese Medicine, Nanchang, Jiangxi, China

**Keywords:** abdominal aortic aneurysm, Sjögren's syndrome, genetic correlations, pleiotropic loci, immune colocalization

## Abstract

**Background:**

Abdominal aortic aneurysm (AAA) and Sjögren's syndrome (SS) frequently coexist, suggesting shared pathogenesis, but their genetic-immunological links are unclear.

**Objective:**

Investigate the shared genetic architecture and immune pathways between SS and AAA.

**Methods:**

Using European GWAS summary statistics (SS: 585 cases/1,546 controls; AAA: 4,083 cases/420,324 controls), we applied complementary genomics approaches: LDSC (genetic correlation), S-LDSC (tissue heritability), PLACO/FUMA (pleiotropic loci), MAGMA/Metascape (pathways), SMR (druggable targets), and HyPrColoc (immune cells).

**Key results:**

A significant positive genetic correlation exists (rg = 0.32, PFDR = 0.021). We identified 8 shared risk SNPs and 6 pleiotropic genes (e.g., HLA-B, HLA-DQB2, LSM2) within loci 6p22.2-21.32. Pathway analyses revealed significant enrichment for MHC class II antigen presentation (*P* = 3.1 × 10^−12^) and U6 snRNA binding/spliceosome (*P* = 2.8 × 10^−7^). Tissue-specific heritability enrichment occurred in artery/aorta, kidney, and secretory tissues (all pS-LDSC < 9.3 × 10^−4^). Immune co-localization implicated myeloid dendritic cells expressing HLA-DR (rs9272318) in convergent dysregulation. HLA-B emerged as a prioritised druggable target (pSMR = 1.65 × 10^−8^).

**Conclusion:**

This study establishes a shared genetic and immunological basis for SS and AAA, driven primarily by dysregulated HLA-mediated antigen presentation (HLA-B/HLA-DR), spliceosome dysfunction, and NK cell impairment. These findings provide mechanistic insights for early AAA detection in SS patients and support developing immunotherapies targeting HLA pathways.

## 1 Introduction

Abdominal aortic aneurysm (AAA) is a focal dilatation of the arterial wall due to weakness caused by atherosclerosis (AS) (1). It is typified by immune cell infiltration, enhanced proteolytic activity, and continuing extracellular matrix component degradation, leading to the aorta wall to dilate (2). AAA is usually asymptomatic and challenging to identify quickly, but once it ruptures, the surgical repair mortality rate can reach 31%–70% (1). Sjogren's Syndrome (SS) is an autoimmune disease (AID) marked by lymphocytic infiltration of salivary and lacrimal glands, leading to glandular dysfunction, where CD4-positive helper T cells (TH) and their cytokines are crucial in the pathogenesis (3).

There are several biological effect mechanisms and possible correlations between AAA and autoimmune-related substances based on evidence from modern medical research.There are significant variations in the quantity of five immune cell types and approximately 40 AID-related bioactive peptides in circulation between AAA and non-AAA patients (4). In AAA patients, IL-1 levels surpass 90 pg/ml (5). Other bioactive peptides, such as myeloperoxidase (MPO) and platelet factor 4 (PF4), additionally display an increasing tendency, surpassing 250 ng/ml and 300 ng/ml, respectively (6). These findings imply that the occurrence of AAA is pathologically impacted by autoimmune diseases (7). Disrupting the connection between Th17 cells and IL-17 can effectively delay the course of AAA, according to studies conducted using mouse models generated by Ang II (8). Additionally, the occurrence of AAA is significantly influenced by neutrophil extracellular traps (NETs), which are secreted by neutrophils. Myeloperoxidase and citrullinated histone H3 (H3Cit), which are indicators of NETs, are markedly increased in AAA patients and have a positive correlation with both MPO and elastase (*P* < 0.001) (9). According to epidemiological research, there is an association between certain AID and AAA.For example, 309 cases (2.0%) of 14,816 patients who underwent elective AAA repair were diagnosed with Rheumatoid Arthritis (RA) during a 13-year cohort study (10). In addition, a 20-year retrospective study discovered that 38.9% of Systemic Lupus Erythematosus (SLE) patients on long-term prednisone treatment may have accelerated AS progression, which might lead significantly to the appearance and death of AAA (11). However, the co-morbidity between SS and AAA has not been studied; hence the purpose of this study was to look at the genetic relationship between SS and AAA.

The concurrent occurrence of AAA and AID consistently encourages research into their shared genetic foundation. Focusing on the proposed, the aim of this study was to investigate the genetic correlation between SS in AID and AAA. However, the impact of AID on immune-related bioactive peptides is the main topic of current study. Based on Lu et al., the induction of autoimmune antigens may be linked to the incidence of AAA (12, 13). The pathological progress in AAA is impacted by this process, which causes the intima to vanish and collagen and elastic fibers to rupture, degrade, and be damaged (14, 15). The effect of inherited telomere length on the correlation between AAA and AID, however, was only investigated by Philip C. Haycock et al. (16). Zhang et al. did not explore the genetic interaction pathways between AAA and SLE, but they did examine the immunological dysregulation based on by genetic parameters, which may be a possible explanation for the co-occurrence of both diseases (17). Relevant routes in AID that are meaningful for the presence, grading, progression, or rupture of AAA (5). It is clear that abnormalities in these processes cause AID and AAA to develop. The urgent need to precisely identify the shared genetic risk loci between SS and AAA. Further clarifying their possible immune-mediated connections is highlighted by considering that there are still substantial knowledge gaps regarding the pleiotropic mechanisms and bidirectional causal relationships between these two diseases. Thus, the purpose of this study was to look into the risk genetic structural loci that SS and AAA share. It is necessary to be aware that the statistical validity of clinical or epidemiological research may face challenges.

**Figure 1:**
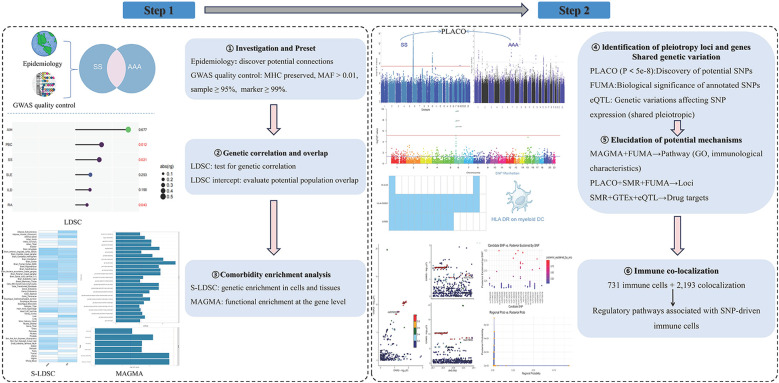
Study workflow: integrated pipeline for shared genetics and immune mechanisms between SS and AAA. (Key steps: LDSC was used to assess genetic correlation; S-LDSC and MAGMA identified cell/tissue and gene-level enrichment; PLACO detected pleiotropic loci, with FUMA annotation; SMR integrated with GTEx prioritized drug targets; HyPrColoc was applied for multi-trait immune colocalization across 731 immune cell traits, identifying 2,193 region–cell colocalization events and highlighting regulatory pathways linking SNPs to immune cell function. AAA, abdominal aortic aneurysm; SS, Sjögren's syndrome; LDSC, linkage disequilibrium score regression; S-LDSC, stratified LDSC; MHC, major histocompatibility complex; MAF, minor allele frequency; PLACO, pleiotropy analysis under composite null hypothesis; FUMA, functional mapping and annotation; eQTL, expression quantitative trait locus; GTEx, Genotype-Tissue Expression; SMR, summary-data-based Mendelian randomization; GO, Gene Ontology; GWAS, genome-wide association study; HyPrColoc, Hypothesis Prioritisation in multi-trait Colocalization).

The following six series of studies were conducted in order to systematically evaluate the genetic overlap, shared susceptibility genes, and potential effector connections between SS and AAA: (1) assessing if there is a genetic association between the two diseases using linkage disequilibrium (LD) score regression (LDSC) based on GWAS summary data (18). (2) The genetic enrichment of SNPs in particular tissues and organs was evaluated using a stratified LDSC (S-LDSC) regression approach. (3) Pleiotropic genetic loci were identified at the SNP level using “PLACO,” risk loci were identified using FUMA, and shared pleiotropic loci were identified using eQTL mapping (19, 20). (4) FUMA, PLACO, MAGMA, and summary data-based MR (SMR) are used to clarify the underlying mechanisms (21). (5) Using immune co-localization to probe immune-mediated processes (22). The various forms of pleiotropy (horizontal or vertical) were then partially explained, and pairwise causal connections were evaluated using the randomization analysis. The flowchart of our study is shown in Figure 1.

## 2 Method

### 2.1 Dataset

GWAS summary statistics for Sjögren's syndrome (SS) were obtained from open-access source: The FinnGen release R11 dataset (4,083 cases and 420,324 controls, all of European ancestry). Abdominal aortic aneurysm (AAA) summary statistics were derived from the GWAS Catalog (GCST012796; 585 cases, 1,546 controls) (23). Genotyping was performed using high-throughput platforms (whole-genome sequencing or SNP microarrays), and standard quality control procedures were applied to remove low-quality samples and variants.

Beagle 4.1 software and the Finnish population-specific SISu v3 reference panel were used for AAA genotype imputation, and the TOPMed reference panel for SS. Eagle 2.3.5 was used for phasing. For eQTL analyses, we used (1) eQTLGen Consortium whole blood cis-eQTL summary statistics (*n* ≈ 31,684) for immune-related expression profiles, and (2) GTEx v8 multi-tissue cis-eQTL data (54 tissues) for tissue-specific regulation. All SNP positions were aligned to GRCh37(hg 19). To avoid potential sample overlap between the SS and AAA cohorts, we verified cohort independence by (i) confirming distinct recruitment sources (FinnGen vs. independent GWAS Catalog datasets) and (ii) cross-referencing with eQTL sample lists. No individual-level overlap was identified between the GWAS and eQTL datasets (Table 1).

**Table 1:** Data source.

Trait	Cases	Controls	Sample size	Resource
Abdominal aortic aneurysm (AAA)	4,083	420,324	424,407	https://r11.finngen.fi/pheno/I9_ABAORTANEUR
Sjögren's syndrome (SS)	585	1,546	2,131	https://www.ebi.ac.uk/gwas/studies/GCST012796

Stringent quality control (QC) procedures were applied to ensure data reliability. Firstly, variants with minor allele frequency (MAF) < 0.01, sample call rate <95%, or SNP call rate <99% were excluded. Post-imputation QC required an INFO score >0.9.

For SMR analyses, cis-eQTLs within ±1 Mb of transcription start sites were tested, with significance defined as p_SMR < 0.05 and p_HEIDI > 0.05 to exclude linkage. MAGMA mapped SNPs to genes ±50 kb using the 1000 Genomes Phase 3 EUR reference panel (24), and gene-based tests were Bonferroni-corrected. PLACO was implemented under the composite null hypothesis using HapMap3 SNPs after LD pruning (r^2^ < 0.1), with significance threshold *p* < 5 × 10^−8^. No individual-level sample overlap existed between the AAA and SS datasets, ensuring unbiased cross-trait analyses.

### 2.2 Quality control standards

We employed the LD Score regression (LDSC) to estimate the genetic correlation between AAA and SS. LD scores were precomputed from HapMap3 SNPs using the 1000 Genomes Project Phase 3 European reference panel (24), matched to the ancestry of the GWAS summary statistics (25). By using the block jackknife method in LDSC, the standard error (SE) was calculated and then adjusted for attenuation bias. Summary statistics were taken from independent sources (AAA: FinnGen; SS: GWAS Catalog), and we confirmed no overlapping individuals across datasets. It is crucial to mention in our study that SS and AAA studies did not exactly overlap in population, which improves the validity of the conclusions. When multiple correlations were tested, Benjamini–Hochberg FDR control was applied with fdr < 0.05.

### 2.3 Organ-level association analysis

We assessed AAA–SS heritability enrichment in specific tissues using Stratified LD Score regression (S-LDSC) (26) with baseline LD model v2.2 and 54 GTEx v8 tissue annotations (27). Enrichment *Z*-scores were computed using default settings with 1000 Genomes EUR LD reference. A Bonferroni-corrected significance threshold of pbon < 0.05/54 was used.

### 2.4 Gene-level exploration and analysis

We mapped overlapping genes according to the lead SNP in each locus in order to investigate the shared mechanisms across AAA and SS. Multi-marker effects were then assessed with Multi-marker Analysis of GenoMic Annotation (MAGMA) (28), mapping SNPs to genes within ±50 kb and using LD from the 1000 Genomes Phase 3 European reference panel. Gene-level significance was controlled by Bonferroni correction across 17,008 genes (pbon < 0.05/17,008 = 2.94 × 10^−6^). For gene-set enrichment, we used MSigDB v7.5.1 curated sets (c2.all) (28, 29, 30). Multiple testing was controlled by Bonferroni across 10,678 tested sets. Pathway enrichment of mapped genes was performed with Metascape (metascape.org) to aid functional interpretation (31). Genome-wide pleiotropic loci identified by PLACO underwent tissue-specific enrichment using GTEx v8 annotations across 54 tissues. We also conducted tissue-specific expression profiling for pleiotropic genes by computing their average transformed expression in each GTEx tissue and classifying genes as up- or down regulated by the sign of the corresponding *t*-statistics.

### 2.5 Pleiotropy analysis and locus validation

We applied Pleiotropy under the Composite null hypothesis (PLACO), a pleiotropy analysis framework under the composite null hypothesis (32), to detect SNPs jointly associated with AAA and SS using a genome-wide significance threshold of *p* < 5 × 10^−8^. Variants were LD-pruned at *r*^2^ < 0.1 based on the 1000 Genomes Project European reference panel. Genome-wide significant SNPs were mapped to genomic risk loci using Functional Mapping and Annotation (FUMA) (33), and shared loci were further evaluated by Bayesian colocalization analysis (34) to assess whether the same causal variant was likely driving both traits. Compared with traditional univariate genome-wide association studies, PLACO increases power to identify shared genetic variants by jointly modeling the null hypotheses for each phenotype, enabling detection of pleiotropic effects even when single-trait associations do not reach genome-wide significance. This approach effectively controls false positives while maintaining sensitivity to loci with modest, concordant effects across traits (32).

### 2.6 Potential exploration of drug targets in European populations

We applied summary-data–based Mendelian randomization (SMR) to integrate summary statistics from genome-wide association studies (GWAS) with expression quantitative trait locus (eQTL) data, in order to identify genes whose expression levels may causally or pleiotropically influence AAA and SS (35). GWAS data were combined with eQTLGen whole-blood and GTEx v8 cis-eQTL datasets using the parameters p_eqtl_smr 5 × 10^−8^ and p_HEIDI 0.05. Only the top cis-eQTL per gene was retained, and associations with HEIDI *p* < 0.05 were excluded. The HEIDI (Heterogeneity in Dependent Instruments) test distinguishes linkage from pleiotropy by assessing heterogeneity among SNP–gene expression associations within a locus (36). This approach increases confidence that observed overlap between GWAS and eQTL signals is due to a shared causal variant, rather than distinct but correlated variants (27). Candidate genes passing these filters were considered supported by multiple lines of evidence, providing insights into regulatory mechanisms underlying the traits.

### 2.7 Immune co-localization analysis

We enhanced the co-localization analysis to find comparable immune-regulatory pathways and processes between SS and AAA by concurrently integrating a broad range of immunological GWAS data (AAA, SS, and 731 immune cell phenotypes) using “HyPrColoc v1.0” (Hypothesis Prioritisation in multi-trait Colocalization) with default priors (prior_prob_coloc = 0.02, prior_prob_single = 1 × 10^−4^) (37). Immune cell GWAS data were sourced from the “GWAS Catalog”. Co-localization significance: “regional probability >0.7” (36, 37). This approach enables hypothesis-free exploration of shared immune mechanisms, although statistical power for rare immune cell subsets (e.g., myeloid dendritic cells) was limited by modest cohort sizes.

## 3 Result

### 3.1 Shared genetic structure between SS and AAA

First, we evaluated genetic correlations between AAA and selected AIDs. Table 2 presents LDSC results. Genetic correlation coefficients (rg) were FDR-corrected. Significant associations (rg > 0, pFDR < 0.05) were reported, accounting for genetic covariance between traits. Results revealed significant genetic associations (*p* < 0.05) between AAA and three AIDs: SS, RA, and primary biliary cholangitis (PBC).

**Table 2:** Genetic correlation results betweenAAA and SS based on LDSC.

Trait pairs	LDSC	
	*r_g_* (SE)	Pfdr
AAA&SS	0.3237	0.0208

LDSC is linkage disequilibrium score regression, SE is standard error, Pfdr is *P*-value adjustment method in the False Discovery Rate.

### 3.2 Results of organ-level association analysis

**Figure 2:**
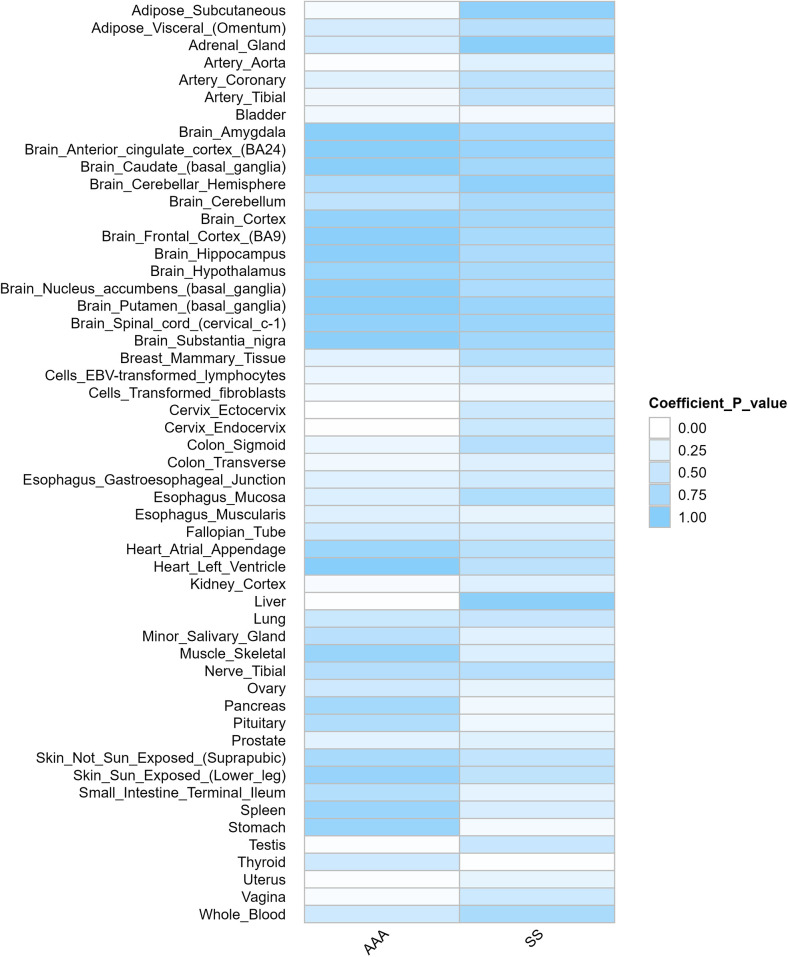
Results of genetic enrichment of traits in different tissues based on S-LDSC.

We employed S-LDSC to assess tissue-specific enrichment of SNP heritability for SS and AAA. First, we obtained GTEx datasets containing gene sets and baseline models for 54 human tissues. Using regression coefficient *Z*-scores and *P*-values, we applied S-LDSC to evaluate tissue-specific genetic enrichment significance. Tissue-specific analysis revealed significant enrichment in multiple tissues including artery, aorta, bladder, kidney, prostate, testis, uterus, and vagina (Figure 2).

### 3.3 Results of gene-level enrichment analysis

**Figure 3:**
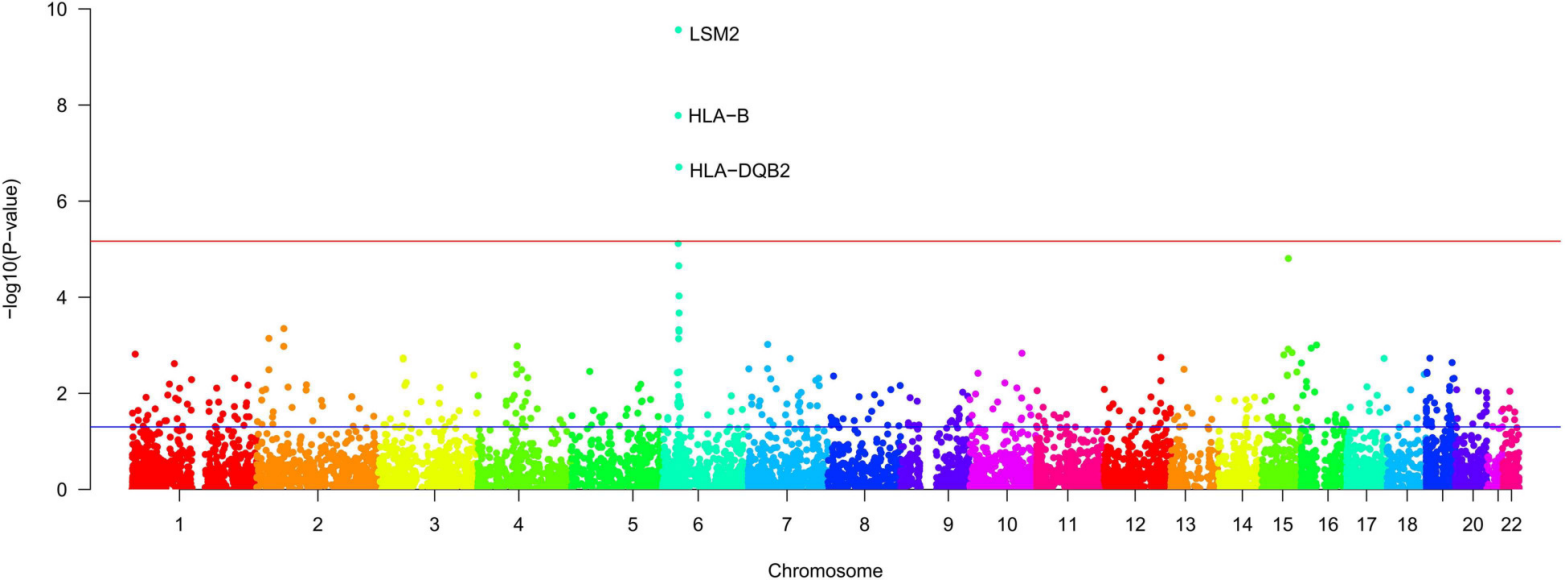
Results of gene enrichment [The *x*-axis represents the chromosomal position, and the *y* axis represents the uncorrected −log10 **(P)** from two-sided *z*-tests for SNP associations with SS. The red line represents a significance threshold of *P* = 5 × 10^−6^].

**Figure 4:**
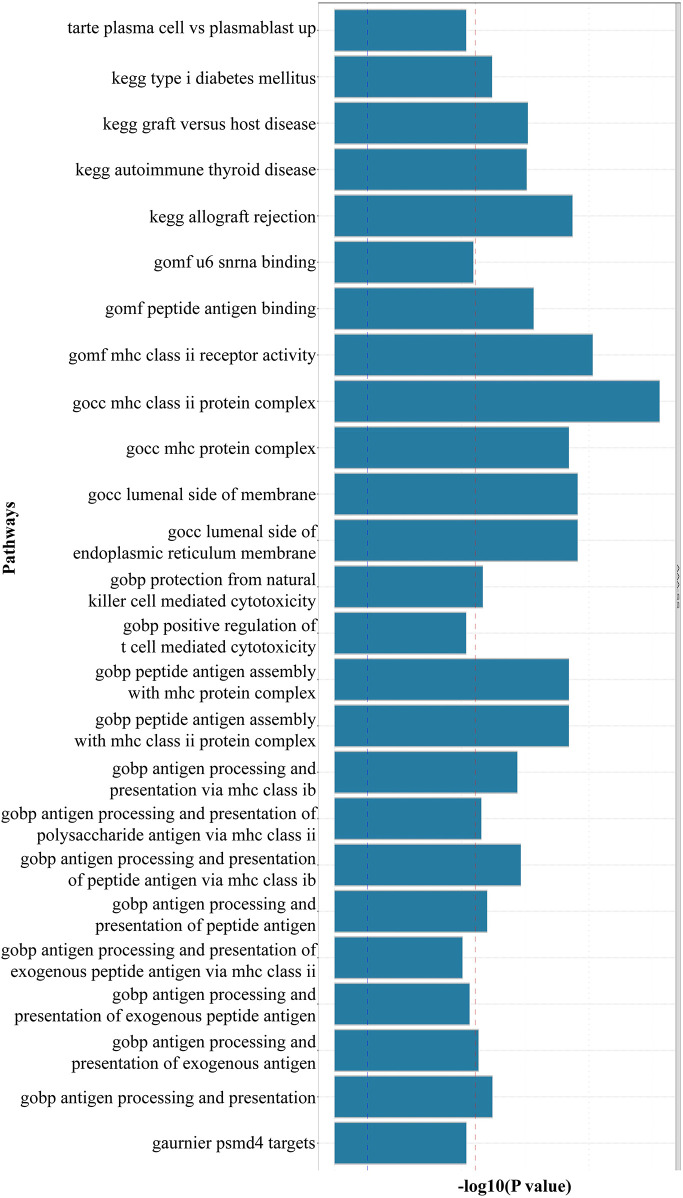
Results of MAGMA gene-set enrichment analysis (the red dotted line indicates the multiple testing-corrected significance threshold of 0.05. Significantly enriched pathways (highlighted in red) include U6 snRNA binding and MHC class II complex, suggesting shared RNA processing and immune activation mechanisms).

**Figure 5:**
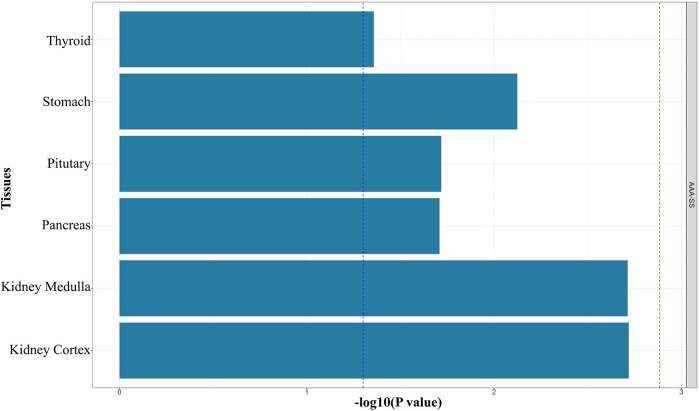
Results of MAGMA tissue-specific enrichment analysis (the red dotted line indicates the multiple testing-corrected significance threshold of 0.05).

Using FUMA for MAGMA gene enrichment analysis, we identified 388 significantly enriched genes associated with SS and AAA. Six genes showed significant enrichment (pfdr><0.05) (Figure 3). These genes participate in key pathways including: allograft rejection; NK cell-mediated cytotoxicity protection; MHC class I/II peptide assembly; antigen processing/presentation; and U6 snRNA binding (Figures 4, 5). Gene set analysis further implicated these genes in pro-inflammatory cytokine secretion, macrophage activation, lysosomal degradation regulation, and snRNP assembly via protein binding. Significant enrichment occurred in secretory tissues including thyroid, stomach, pituitary, pancreas, kidney medulla, and kidney cortex, suggesting SS-mediated disruption of secretory functions. However, tissue specificity did not survive Bonferroni correction for 54 tests (pb > 0.05/54). These results demonstrate shared SS-AAA genetic mechanisms.

### 3.4 Results of SNP-level pleiotropy evaluation and risk loci analysis

Based on LDSC-identified shared genetics, we applied pleiotropy analysis (PLACO) to detect pleiotropic loci. Using PLACO results, we employed FUMA to identify AAA-associated SS risk loci (Table 3). Notably, shared pleiotropic regions (e.g., 6p22.2, 6p21.33, 6p21.32) were identified for both SS and AAA (Supplementary Table S1).

**Table 3:** Pleiotropic genomic risk loci between AAA and SS.

uniqID	rsID	Locus boundary^a^	pos	p	nSNPs	nGWASSNPs	IndSigSNPs^b^	LeadSNPs^c^
6:26426628:A:G	rs2076030	6:26,349,951–26,426,628	26,426,628	4.55 × 10^−6^	2	1	rs2076030	rs2076030
6:31376571:C:T	rs3957110	6:30,455,161–31,376,571	31,376,571	1.50 × 10^−6^	5	1	rs3957110	rs3957110
6:32636282:A:G	rs9272318	6:31,765,409–33,642,368	32,636,282	1.01 × 10^−11^	1924	16	rs3135391; rs9271583; rs9268581; rs9270806; rs9272318; rs9275379; rs9275439; rs2859124	rs3135391; rs9271583; rs9272318; rs9275379; rs9275439; rs2859124

^a^ Locus boundary of each pleiotropic genomic risk locus was denoted as “chromosome: start-end” defined by FUMA for the corresponding trait pair.

^b^ IndSigSNPs usually refer to Independent Significant SNPs, meaning that after removing the correlation, the remaining SNPs that are independently and significantly associated with the disease will be identified as IndSigSNPs.

^c^ Lead SNPs are the most significant single nucleotide polymorphisms in GWAS, the signature SNPs that are most associated with a trait or disease.

### 3.5 Drug targets in European populations

**Figure 6:**
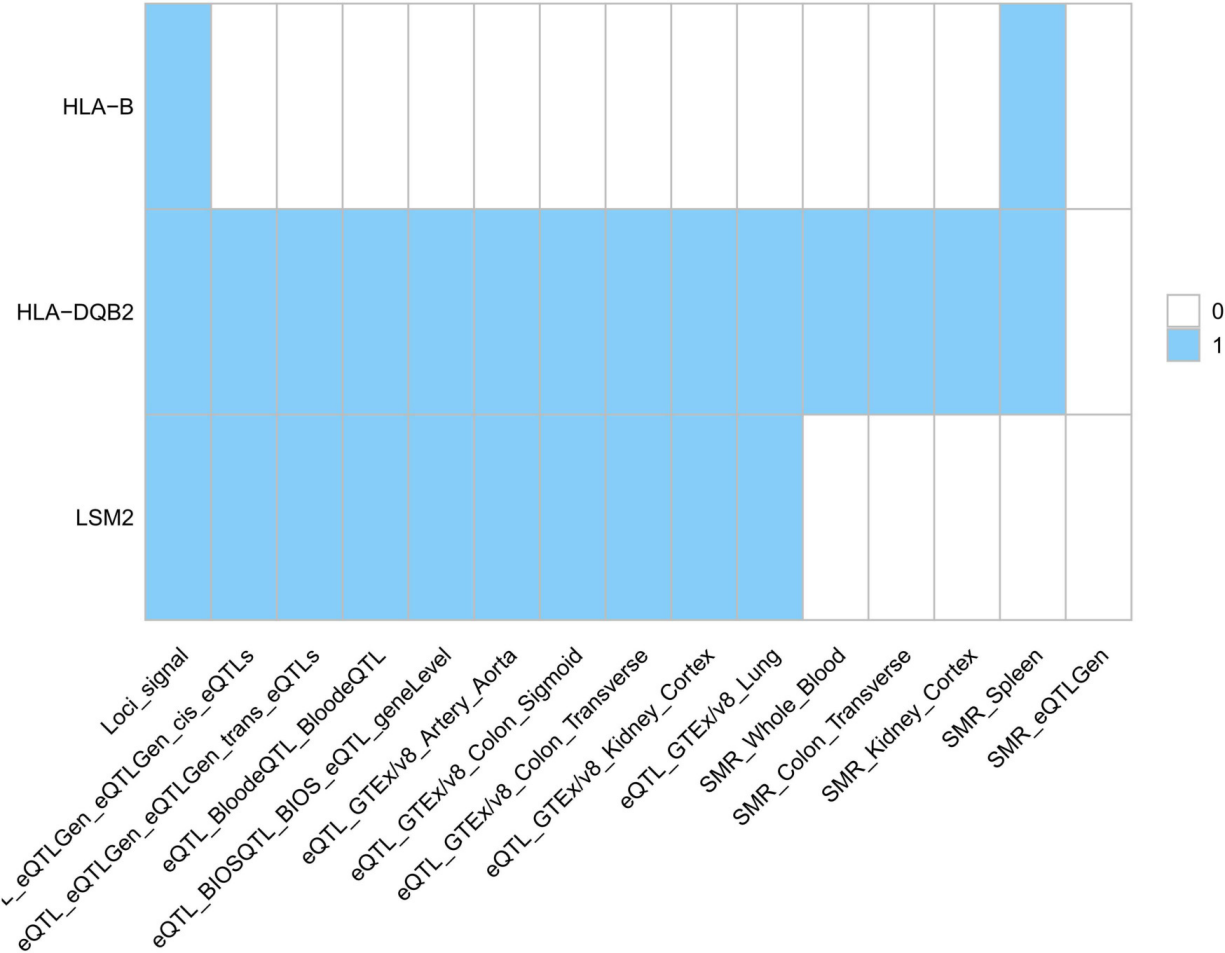
European population drug target (signals represent gene hits across trait pairs. eQTL, expression quantitative trait locus; SMR, summary-data-based Mendelian randomization).

First, we identified three potential drug targets using SMR (p_SMR < 0.05, p_HEIDI > 0.05, p_b < 0.05/15,638 = 3.2 × 10^−6^) (Figure 6). While HLA-B mediates organ transplantation compatibility (38), HLA-DQB2 is implicated in metastatic lung adenocarcinoma (39), and LSM2 in hepatocellular carcinoma (HCC) (40), these loci lack established roles in AAA or SS pathogenesis. We integrated PLACO, FUMA, MAGMA, and SMR results to identify pleiotropic genes associated with multiple traits (Table 4). These genes showed significant tissue-specific genetic signals. eQTL and SMR analyses confirmed their pleiotropic effects across traits and provided chromosomal localization.

**Table 4:** European population drug target results.

SYMBOL	GENE	Locus boundary^a^	NSNPS	NPARAM^b^	N	ZSTAT	P	Pbon	sig.placo^c^	SMR_Whole_Blood	SMR_eQTLGen	eQTL_	band^d^
HLA-B	ENSG00000234745	6:31,321,649–31,324,965	8	5	442,405	5.525	1.65 × 10^−8^	0.00012319	1	0	0	0	p21.33
HLA-DQB2	ENSG00000232629	6:32,723,875–32,731,311	1	1	442,405	5.0732	1.96 × 10^−7^	0.001462081	1	1	0	1	p21.32
LSM2	ENSG00000204392	6:317,651,735–31,774,761	1	1	442,405	6.2057	2.72 × 10^−10^	2.04 × 10^−6^	1	0	0	1	p21.33

^a^ Locus boundary of each pleiotropic genomic risk locus was denoted as “chromosome: start-end” defined by FUMA for the corresponding trait pair.

^b^ NPARAM is the number of variables considered in Mendelian Randomization studies.

^c^ The sig.placo marks whether the gene or SNP shows significant correlation in a particular phenotype or whether it is located in a functionally important region.

^d^ The band refers to a genomic regional banding zone that may indicate the chromosomal region or banding interval in which its genes or SNPs are located.

### 3.6 Results of immuno-colocalization

**Figure 7:**
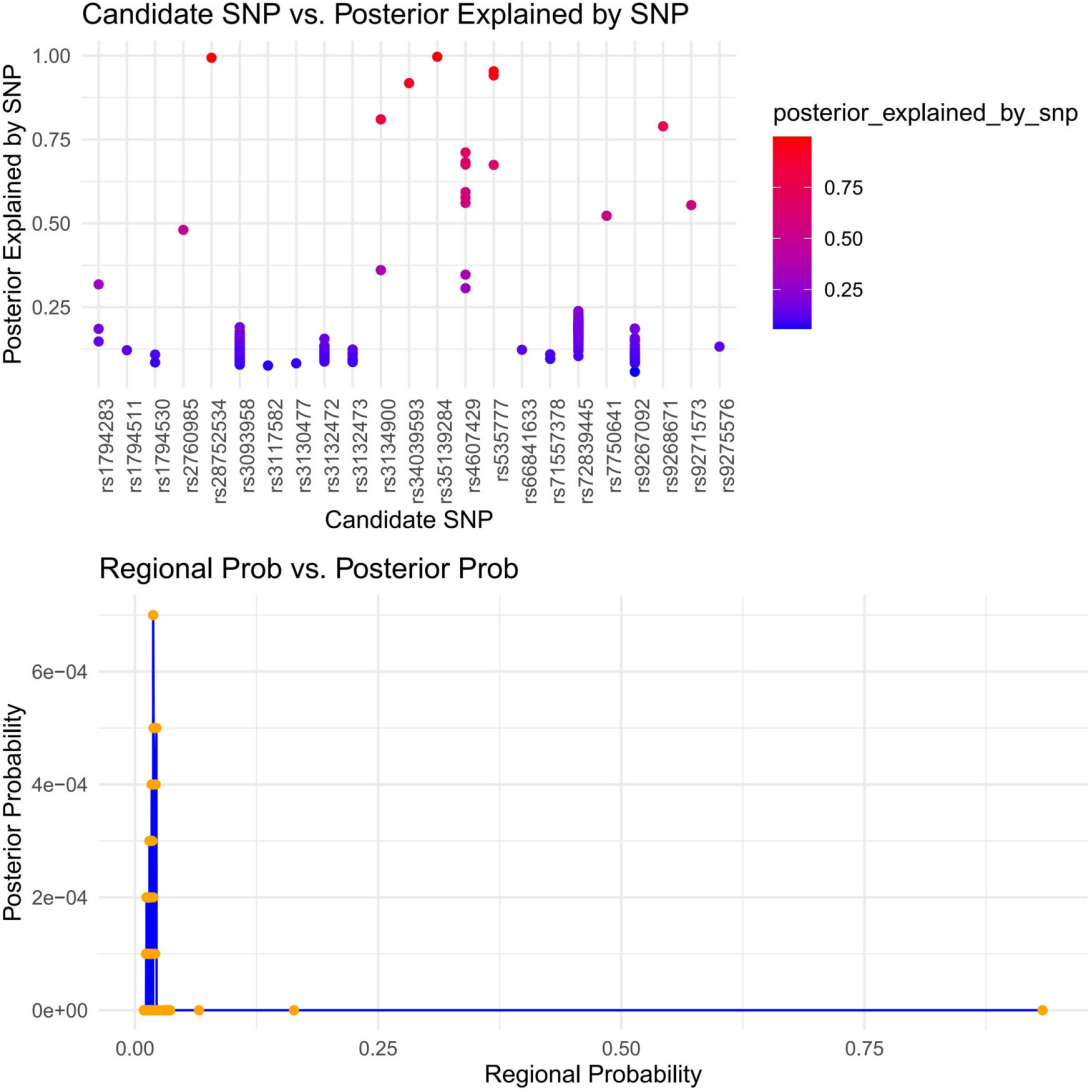
Immune colocalization analysis of AAA and SS risk loci with immune cell traits using hyPrColoc. (HyPrColoc (Hypothesis Prioritisation in multi-trait Colocalization) was applied to three pleiotropic loci identified by PLACO for AAA and SS, testing colocalization with 731 immune cell phenotypes. The top panel shows the SNP-level posterior probability of being the shared causal variant for each candidate SNP. The bottom panel compares regional posterior probability (*x*-axis) and SNP-level posterior probability (*y*-axis) across all locus–cell pairs. The vertical reference line at a regional posterior probability of 0.7 indicates the threshold for significant colocalization. Points to the right of this line represent AAA–SS–immune trait combinations with strong evidence of a shared causal variant).

Immune-mediated AAA pathogenesis is suggested by shared dysfunction in secretory tissues (thyroid, stomach, pituitary, pancreas, renal medulla/cortex). We applied HyPrColoc to identify immune cells showing multi-trait co-localization. This analysis targeted critical immune cells (Supplementary Table S2). One immune cell type showed significant contribution to SS, supported by a multi-effector locus with shared causal variants (*p* > 0.6). We confirmed conventional dendritic cells (cDCs) significantly influence specific immune subsets. Notably, myeloid dendritic cells showed AAA-associated HLA-DR expression driven by rs9272318, suggesting mechanistic links between secretory dysfunction and immune processes (Figure 7).

## 4 Discussion

SS is a systemic autoimmune disorder affecting multiple organ systems. AAA involvement in SS may lead to severe complications. We applied complementary analytical approaches with distinct modeling frameworks to comprehensively investigate pleiotropic relationships.

LDSC identified significant genetic associations between AIDs and AAA, specifically SS, RA, and PBC. Although RA and PBC comorbidity with AAA is established (10, 41, 42), SS-AAA evidence was previously confined to a single case report (43). We provide the first population-level genetic evidence of shared SS-AAA genetic architecture. This shared risk likely arises from immune dysregulation, demonstrated by T cell, B cell, and antigen-presenting cell infiltration in AAA lesions (12)—key features of SS pathogenesis.

### 4.1 Multi-omics approach revealed key shared biological mechanisms

Antigen Presentation & MHC Pathways: Gene enrichment analysis revealed MHC class II pathway involvement (Figure 4). Immunolocalization identified HLA-DR+ myeloid dendritic cells as a key shared signature (regional probability >0.6; Figure 7). This convergence implies aberrant antigen presentation activates autoreactive T cells in both diseases (44, 45), evidenced by HLA-DR+ dendritic cell enrichment in AAA lesions (12) and SS salivary glands (46).

Spliceosome Dysregulation: U6 snRNA binding pathway enrichment (Figure 4) indicates spliceosome dysfunction as a novel link. This connects autoimmunity to ECM degradation—a feature of SS (glandular disruption) (47) and AAA (aortic rupture via collagen/elastin defects) (2, 48).

NK Cell Dysfunction: Enrichment of NK cell-mediated cytotoxicity (Figure 4) and lead SNP rs2076030 in the KIR locus (6p22.2; Table 3) implicate NK dysfunction as a shared mechanism. In SS, impaired NK surveillance permits autoantibody production (46), whereas in AAA, defective cytotoxicity fails to control macrophage-driven inflammation (12). This KIR/HLA imbalance constitutes a regional pathogenic risk.

Shared genomic loci (6p21.32-33, 6p22.2; Table 3, Supplementary Table S1) overlap regions associated with autoimmune diseases, including rheumatoid arthritis (RA; rs2076030 at 6p22.2) (49). These loci contain genes regulating immune activation, antigen presentation, and inflammation (50, 51). Tissue-specific heritability enrichment (S-LDSC; Figure 2) occurred in immune-relevant (artery, aorta) and secretory tissues (bladder, kidney, prostate, testis, uterus, vagina). This parallels SS-related secretory gland and renal/bladder dysfunction (52), suggesting systemic inflammation (53, 54, 55) or immune-metabolic injury (56, 57) links SS secretory defects to AAA vascular vulnerability. Artery/aorta enrichment indicates direct vascular immune involvement, potentially through hemodynamic stress- or IFN-γ-induced vascular smooth muscle cell (VSMC) inflammation (58).

### 4.2 Clinical translation and novel therapeutic insights

Drug target analysis identified HLA-B, HLA-DQB2, and LSM2 as druggable targets (Table 4, Figure 6). HLA-B emerges as a particularly promising candidate. Its role in presenting antigenic peptides to activate self-reactive T cells (59, 60) is central to SS pathogenesis and is now implicated in AAA susceptibility. However, the direct therapeutic targeting of specific HLA molecules remains challenging due to their extreme polymorphism and fundamental role in adaptive immunity. Consequently, no clinical trials are currently running that directly target HLA-B or HLA-DR alleles for SS or AAA. However, broader immunomodulatory strategies that indirectly impact HLA-mediated antigen presentation, such as T-cell co-stimulation blockade (e.g., with Abatacept) or Janus kinase (JAK) inhibition, are being explored in related autoimmune contexts and represent a promising indirect avenue for translation (61, 62). The colocalization of rs9272318 with HLA-DR on myeloid DCs further implicates antigen presentation as a convergent and therapeutically targetable node. These findings provide a strong genetic rationale for exploring immunomodulatory approaches (63). This could involve repurposing existing therapies or developing novel biologics targeting these shared pathways, enabling early AAA intervention and concurrent management of SS-AAA comorbidity (3).

### 4.3 Molecular mechanistic conclusion

In summary, our integrated genetic analyses delineate a convergent pathogenic model whereby shared risk loci (e.g., HLA-B, HLA-DQB2) primarily drive the comorbidity of SS and AAA through a core mechanism of dysregulated antigen presentation. This process activates autoreactive T cells and initiates a chronic inflammatory cascade (64). While fundamentally immune-mediated, this inflammation secondarily triggers the activation of vascular endothelial and smooth muscle cells within the aortic wall, culminating in the extracellular matrix degradation and remodeling that defines AAA progression (65). Thus, the interplay between innate immune dysregulation and subsequent vascular cell activation forms the critical bridge connecting systemic autoimmunity to local aortic wall instability (66, 67).

## 5 Limitation

While our study identified shared genetic architecture between SS and AAA in European populations, the generalizability of these findings to diverse populations requires further validation. Future studies should prioritize multi-ethnic cohorts to evaluate the relevance of identified risk loci (including HLA-B and LSM2) and immune pathways across populations. Population stratification analyses were limited by our use of summary-level data. GWAS data for specific immune cell subsets (e.g., HLA-DR + myeloid dendritic cells) had limited sample sizes, potentially reducing the robustness of colocalization signals such as rs9272318-HLA-DR. Leveraging larger immunogenomic resources (e.g., UK Biobank) and performing single-cell eQTL mapping in diseased tissues will be essential. Concurrently, single-cell multi-omics expansion strategies and AI-enhanced phenotypic analysis (developing deep phenotypic inference models: predicting the proportions of 78 immune cell subpopulations using conventional blood test parameters; establishing a transfer learning framework: utilizing mouse model data to enhance human GWAS signal detection) can improve statistical power and result reliability. Three-dimensional genome-guided localization (integrating Hi-C/ChIA-PET data to construct immune cell-specific chromatin interaction networks) and cross-omics Bayesian integration models can further achieve closed-loop validation from genetic signals to functional mechanisms. Although we propose mechanistic links (HLA-DR+ dendritic cells, U6 snRNA processing, KIR-HLA interactions) supported by genetic colocalization and pathway enrichment, experimental validation is required: 1) validate spatially resolved HLA-DR expression and U6 snRNA-associated splicing factors in tissues from patients with SS-AAA comorbidity; 2) assess NK cell function in carriers of identified KIR variants with both SS and AAA.

## 6 Conclusion

Our study reveals significant genetic and immunological crosstalk between SS and AAA. We identified shared risk loci in chromosomal regions 6p22.2, 6p21.33, and 6p21.32, implicating three convergent mechanisms: 1) dysregulated antigen presentation by HLA-DR^+^ myeloid dendritic cells; 2) spliceosome dysfunction involving U6 snRNA-binding factors; 3) impaired natural killer (NK) cell function associated with KIR-HLA interactions. Druggable targets, particularly HLA-B, were identified, supporting development of novel immunotherapies concurrently targeting both disorders. These findings provide critical insights for: 1) early AAA detection in SS cohorts; 2) concurrent management of SS-AAA comorbidity.

## Data Availability

The original contributions presented in the study are included in the article/Supplementary Material, further inquiries can be directed to the corresponding author.
